# Prevalence of stunting and its associated factors among children 6-59 months of age in Libo-Kemekem district, Northwest Ethiopia; A community based cross sectional study

**DOI:** 10.1371/journal.pone.0195361

**Published:** 2018-05-03

**Authors:** Selamawit Bekele Geberselassie, Solomon Mekonnen Abebe, Yayehirad Alemu Melsew, Shadrack Mulinge Mutuku, Molla Mesele Wassie

**Affiliations:** 1 Program Development Division, World Vision Ethiopia, Addis Abeba, Ethiopia; 2 Department of Human Nutrition, Institute of Public Health, College of Medicine and Health Sciences, University of Gondar, Gondar, Ethiopia; 3 Department of Epidemiology and Biostatistics, Institute of Public Health, College of Medicine and Health Sciences, University of Gondar, Gondar, Ethiopia; 4 Adelaide Medical School, Faculty of Health and Medical Sciences, University of Adelaide, Adelaide, Australia; Quensland University of Technology, AUSTRALIA

## Abstract

**Background:**

Children in developing countries are highly vulnerable to impaired physical growth because of poor dietary intake, lack of appropriate care, and repeated infections. This study aimed at assessing the prevalence of stunting and associated factors among children 6–59 months of age in Libo-kemekem district, northwest Ethiopia.

**Methods:**

A community based cross sectional study was conducted in Libo-Kemekem from October 15 to December 15, 2015. The multistage sampling technique was employed to select 1,320 children aged 6-59months. Data were collected by trained community health extension workers under regular supervision. Data were entered into EPI-Info version 3.5.1, and height for age was converted to Z-score with ENA-SMART software. Data were then exported to SPSS version 20 for descriptive and binary logistic regression analysees. The significance of associations was determined at p<0.05.

**Results:**

Out of 1287 children included in the analysis, 49.4% (95% CI: 46.7%–52.3%) were found to be stunted. In the multivariate analysis, increased child age [AOR = 6.31, 95%CI: (3.65, 10.91)], family size of six and above [AOR = 1.77, 95%CI: (1.35, 2.32)] were positively associated with stunting, while, fathers with secondary school education [AOR = 0.50, 95%CI: (0.30, 0.81)], farmers as household heads [AOR = 0.56, 95%CI: (0.38, 0.84)] and self-employed parents as household head [AOR = 0.45, 95% CI: (0.28, 0.72)] were found to be preventive factors.

**Conclusion:**

The prevalence of stunting was high in the study area. We found that stunting was significantly correlated with child age, occupational status of household head, family size, and fathers’ education. Therefore, intervention focusing on supporting housewives, family planning, and education on child feeding and nutrition should be implemented.

## Background

Stunting is defined as a height that is more than two standard deviations below the World Health Organization (WHO) child growth standard median [1, 2]. Stunting is considered as a severe public health problem in the community when its prevalence in children is greater than 40% [3]. It is a largely irreversible outcome of inadequate nutrition and repeated bouts of infection during the first 1000 days of the child’s life [1, 4]. It has long term effects on individuals and societies, including diminished cognitive and physical development, reduced productive capacity, and poor health, and increased risk of degenerative diseases such as diabetes [[4, 5]. Furthermore, stunted children experienced rapid weight gain after 2 years have an increased risk of becoming overweight or obese later in life [4, 5].

Globally 161 million children under five were stunted in 2013 [6]. In 2015, Africa has the highest prevalence of stunting at 37.6%, followed by Asia at 22.9% [7] According to the Ethiopian mini Demographic and Health Survey (EDHS) report 2014, stunting among children under five years of age is at 40%. In the Amhara National Regional State of Ethiopia stunting, wasting, and underweight is reported to be 40%, 10% and 33%, respectively [8].

Stunting can be caused by various factors such as parental, socio-demographic, and economic status, as well as cultural practices and environmental and other health related variables [9]. For instance, poverty, low parental education, lack of sanitation, low food intake, poor feeding practices, inadequate breastfeeding, repeated infections, family size and birth interval are regarded as key determinants of stunting [9–11]. Another study reported that family socio-economic status was the most important factor associated with stunting [12]. Similarly, other studies are in agreement that stunting is influenced by child age [13], age of the mother, child sex, family size, wealth index [13], maternal/paternal education, marital status of mother, and number of livestock of the family [10, 14–19]. Moreover, availability and utilization of health services and the care provided to the child were found to be other determinants of stunting [20].

The Ethiopian government recognizes stunting as a major public health problem and obstacle to its economic goals. Since stunting is greatly dependent on the local geo-cultural factors such as tradition and community livelihood, investigating its prevalence and causative factors within this context is important to prioritize development interventions to mitigate the problem. Therefore, the aim of this study was to determine the magnitude of stunting and identify its determinants among children aged less than five years in Libo-kemkem district, northwest Ethiopia.

## Methods

### Study design and setting

A community based cross sectional study was conducted in Libo-Kemkem district from October 15 to December 15, 2015, to determine the level of stunting among children 6–59 months of age. The district has an area of 1,560 km^2^ and is located at 11°57’46.6’-12°25’32.6N latitude and 37°34’48.9–38°3’30.9” E longitude. It comprises 34 villages of which 5 are urban. The district is located on black cotton clay soil and flat plain with relatively high temperature and high rainfall, with a mean of 1173mm rain per annum. Agricultural activities are restricted to a single rain season (from June to September). Maize, barley and millet are the main food crops, while rice, vetch, and chickpeas are the main cash crops. The total population of the district in 2010 was 198,951 of which 100,951 were males and 97,423 females. The district has a population density of 1948 per square km [21].

### Ethics approval

Ethical clearance was obtained from the Ethical Review Committee of the Institute of Public Health, College of Medicine and Health Sciences, the University of Gondar. Letters of permission were also obtained from the North Gondar Zonal Health Office and the Libo-kemekem District Administration. Informed consent obtained from each parent/care giver after the purpose of the study was explained. Confidentiality was ensured by using code numbers rather than names.

### Study population and sampling

The study population included children aged 6–59 months in the 12 randomly selected villages of the district, three urban and nine rural villages. Children who were seriously ill during the whole data collection season and children with spinal curvature (Kiphosis, scoliosis and kiphoscoliosis) were excluded. Out of 34 villages in the district, 12 were selected randomly. The total sample size (n = 1320) was distributed to each village proportionally based on the number of households in the village, using probability proportionate to size method. The number of households in each village was obtained from the respective health posts. Sampling interval (*K*) was calculated for each village, and the first household in each village was identified using a random number from *k* number of households. Then, systematic random sampling technique was used to select study participants from selected households for measurements. For households which had more than one eligible children, lottery method was used to select one child for the study. Mothers or care givers were interviewed on socio-demographic, economic, child health related characteristics and environmental conditions with a pre-tested structured questionnaire. Child morbidity status was asked in the previous 6 months as diagnosed by a health professional.

### Data collection and analysis

Data were collected by trained community health extension workers from October 15 to December 15, 2016. Mothers or care givers were interviewed and anthropometry measurement (height and weight) was taken on children.

Height of infants aged six months to 23 months was measured in a recumbent position to the nearest 0.1 cm, using a board with an upright wooden base and movable headpieces. Children aged 24 to 59 months were measured in a standing up position to the nearest of 0.1 cm. Additionally, child weight was measured by an electronic digital weight scale for children who were comfortable to be measured alone, and also for children who were uncomfortable to be measured alone, we used the combined mother and child weight and the mother’s individual weight to calculate the child’s weight [3].Respondent economic status was accounted for through the occupational variables. The Categories of morbidity status were based on the types of diseases that the child encountered in the previous six months. For instance, if the child had one type of disease it will be categorized as one disease. Distance of water source from household was categorized as near if it takes less than 30 minutes while far if it takes ≥30 minutes on foot.

The collected data underwent cleaning and entered using the EPI-INFO 3.5.1 software. Data on sex, age, height, and weight was transferred with participants’ identification number to ENA for SMART software to convert nutritional data into Z scores of the indices HAZ using the WHO standard. The anthropometry measurement of height for age (HAZ) was calculated through ENA SMART software, and children less than -2 SD were classified as stunted. Those children with HFA indices between -2 and -3 SD were classideid as moderate stunting while < -3 SD were classified as severe stunting. Data was also exported to SPSS version 20 for further analysis and identification of factors associated with stunting by the binary logistic regression model. Variables with a p-value less than 0.2 in the bivariate analysis were included in the multivariate logistic regression model. The strength of association was determined by the Adjusted Odds Ratio (AOR) at a 95% confidence interval, and p-value <0.05 was used to show the association between independent variables and the presence of stunting. Variables having p-value, of < 0.05 were considered as statistically significant.

## Results

### Demographic and socio-economic characteristics

In this study a total of 1287 children aged 6-59months were included, with a response rate of 97.5%. The majority 1149 (86.9%) of the mothers were married, and 788 (61.2%) were within the age group of 26–35 years. With regard to parents educational status, 61.2% of the mothers and 47.6% of the father were illiterate. Out of the total households included, 649 (53.9%) family heads were farmers. **(Table 1)**

**Table 1:** Demograpic and socio-economic characteristics of parents in Libo-kemekem district, northwest Ethiopia.

Characteristics	Category	Frequency	Percent (%)
Age of the mother	18–25	278	21.6
	26–35	788	61.2
	36 and above	221	17.2
Marital status*	Single	43	3.3
	Married	1149	86.9
	Divorced, Widow and separated	125	9.7
Husband education	Cannot read & write	612	47.6
	Primary education	432	33.6
	Secondary education	111	8.6
	Tertiary education	132	10.3
Maternal education	Cannot read & write	788	61.2
	Primary education	309	24.0
	Secondary education	104	8.1
	Tertiary education	86	6.7
Occupational status of head of the HHs	House wife	237	18.4
	Farmer	694	53.9
	Merchant	115	8.9
	Government employee	66	5.1
	Self-employee	175	13.6

*n is not 1,287.

The children varied in terms of sex and age in that 665 (51.7%) were females, while 367 (28.5%) and 356 (27.7%) were 13–25, and 25–36 months old, respectively. Regarding child morbidity status, most of the children 948 (73.7%) had infectious diseases such as diarrhea caused by infectious agents for in the previous six months. **(Table 2)**

**Table 2:** Children health and characteristics at Libo-kemekem district, northwest Ethiopia.

Characteristics	Category	Frequency	percent
Sex of child	Male	622	48.3
	Female	665	51.7
Age of child	0–12	159	12.4
	13–24	367	28.5
	25–36	356	27.7
	37–48	284	22.1
	49–59	121	9.4
Morbidity status*	Only 1 disease	138	10.7
	Only 2 diseases	948	73.7
	3 and above diseases	201	15.6

* The categories of morbidity status were based on the types of diseases that the child encountered in the previous six months.

### Environmental health condition

The majority (71.3%) of the households used public tap water for drinking. Almost all, 1170 (90%), of the households had access to a nearby water source, whereas 117 (9.1%) are required to travel more than 30 minutes on foot to fetch water.

With regard to the availability of toilet, 745 (57.9%) households had toilettes; traditional pit latrines were most commonly used, whilst 529(41.1%) households used open field defecation. **(Table 3)**

**Table 3:** Environmental health conditions of households in Libo-kemekem district, northwest Ethiopia.

Characteristics	Category	Frequency	Percent
Availability of toilet*	Yes	745	57.9
	No	529	41.1
Source of water*	River	156	12.1
	Spring	62	4.8
	Public tab	917	71.3
	Others	74	5.7
Distance of water source	Near	1170	90.9
	Far	117	9.1

*n is not 1287.

## Prevalence of stunting

**Fig 1:**
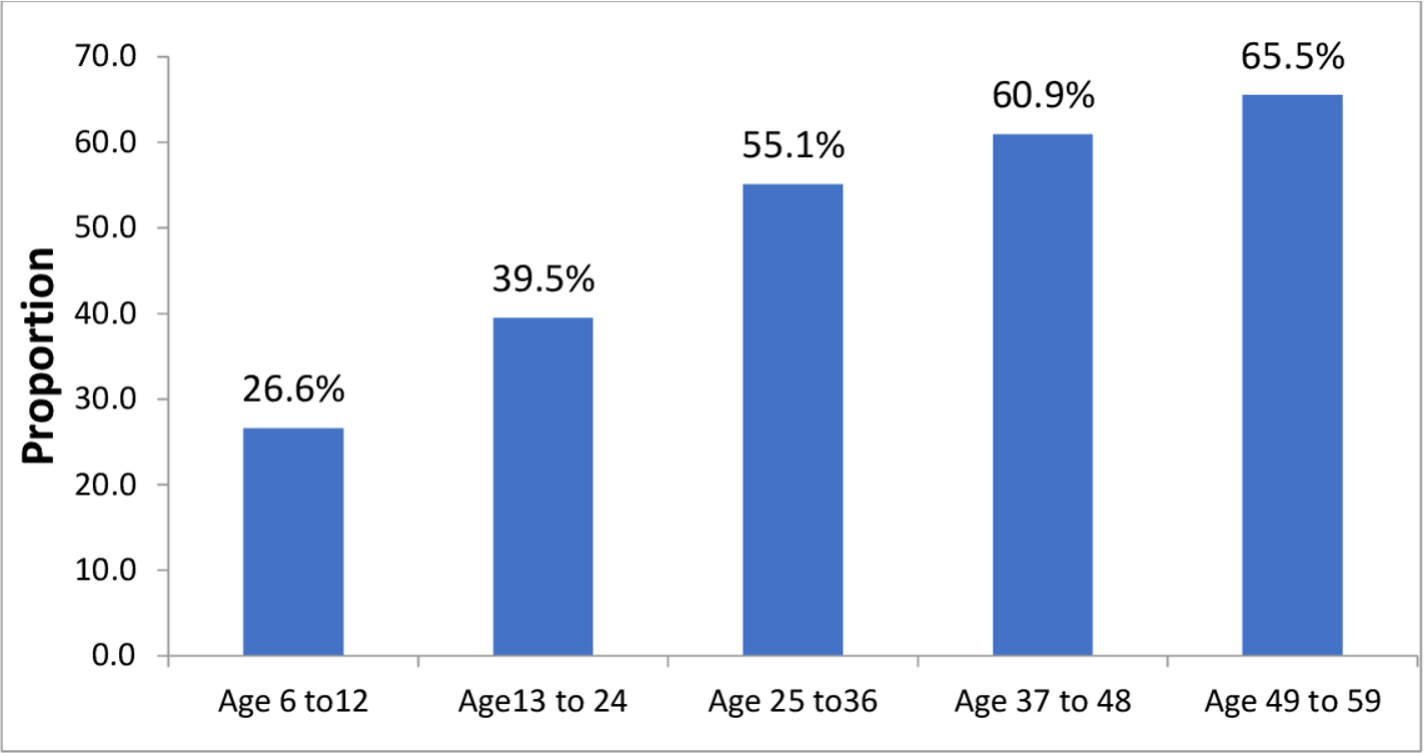
Prevalence of stunting by age (in months) among children aged 6–59 months at Libo-kemekem district, northwest Ethiopia.

The overall prevalence of stunting in the study population was 49.4% [95% CI: 46.7–52.3]. The prevalence of stunting was 52.3% among female children and 47.7% among males. The prevalence of moderate and severe stunting was 37.5% and 13.1%, respectively. Stunting was most prevalent in the 49–59 months age group at 65.5%, while the 6–12 months age group had the least. (Fig 1)

### Factor associated with stunting

Child age, family size, fathers educational status, occupational status of household head, child morbidity status, and marital status of parents were entered into the multivariate binary logistic regression model. The output of the multivariate binary logistic regression showed that, child age, family size, fathers educational, and occupational status were significantly associated with stunting. **(Table 4)**

**Table 4:** Binary logistic regression analysis output for factors associated with stunting among children age 6-59months, in Libo-kemekem district, northwest Ethiopia.

Variables	Stunting		Crude OR (95% CI)	Adjusted OR (95% CI)
	Yes	No		
**Morbidity**				
Only Diarrhea	64	73	1	1
Diarrhea & ARI	439	496	1.010(0.705,1,446)	0.84(0.57,1.25)
Diarrhea,ARI & other diseases	125	75	1,901(1.223,2.955)	1.46(0.90,2.36)
**Age of child**				
6–12 months	42	116	1	1
13–24 months	143	219	1.803 (1.196,2,720)	2.07(1.34,3.18)*
25–36 months	195	159	3.387(2,247,5,106)	3.86(2.50,5.97)*
37–48 months	170	109	4.308(2,810,6,603)	4.73(3.00,10.91)*
49–59 months	78	41	5.254(3.132,8,813)	6.31(3.65,10.91)*
**Number of family size**				
Less than and equal to 5	346	450	1	1
6 and above	282	194	1.891(1.502,2.380)	1.77(1.35,2.32)*
**Marital status**				
Single	25	18	1	1
Married	541	564	0.691(0.373,1.280)	0.62(0.32,1.23)
Divorced, separated and widowed	62	62	0.720(0.357,1.451)	0.94(0.43,2.03)
**Father’s educational status**				
Cannot read and write	336	266	1	1
Primary education (1–8)	200	227	0.698(0.544,0.895)	0.75(0.57,1.00)
Secondary education (9–12)	38	73	0.412(0.270,0.630)	0.50(0.30,0.81)*
College and above	54	78	0.548(0.374,0.803)	0.63(0.36,1.11)
**Occupational status of household head**				
House wife	125	110	1	1
Farmer	348	334	0.917(0.681,1.234)	0.56(0.38,0.84)*
Merchant	55	60	0.807(0.516,1.265)	0.67(0.41,1.10)
Government employed	25	41	0.537(0.307,0.939)	0.68(0.34,1.37)
Self-employed	99	75	0.667(0.449,0,989)	0.45(0.28,0.72)*

*Statistically significant at p-value less than 0.05

Age of a child was directly correlated with stunting. Accordingly, compared to children aged 6-12months, children of age13-24 months were 2.07 times more likely to be stunted [AOR = 2.07, 95% CI: (1.34, 3.18)]. Similarly, children aged 25–36 months had 3.86 times more odds of being stunted than children aged 6-12months [AOR = 3.86, 95%CI: (2.50, 5.97)]. Thus older children had a stronger association with stunting. Children aged 37–48 months were 4.73 times [AOR = 4.73, 95%CI: (3.00, 10.91)] more stunted while children aged 49–59 months were 6.31 times more likely to be stunted compared to children aged 6-12months [AOR = 6.31, 95%CI: (3.65, 10.91)].

Family size had also shown a positive significant association with stunting. Children in a family of at least six members were 1.77 times at higher odds of stunting than children in a family of five and less [AOR = 1.77, 95%CI: (1.35, 2.32)].

Reduction in the odds of stunting was observed among children who lived with their fathers and whose parents were farmers and self-employed. Children whose fathers completed secondary school education had shown 50% reduced odds of being stunted compared to children with illiterate fathers [AOR = 0.50, 95%CI: (0.30, 0.81)]. Similarly, farmers and self-employed household heads reduced the odds of their children compared to housewife heads. As a result children of farmer household heads had 44% lower odds of being stunted [AOR = 0.56, 95%CI: (0.38, 0.84)] than children of housewives. Similarly, children from self-employed household heads had 55% lower odds of stunting than housewives [AOR = 0.45, 95% CI: (0.28, 0.72)].

## Discussion

This study has explored the prevalence of stunting and its associated factors among children aged 6–59 months at Libo-kemekem district, North West Ethiopia. The prevalence of stunting was 49.4%, of this 47.3% in males and 50.3% in females. This finding was the highest compared to the regional, national and WHO cut off point of 40% set for stunting [8, 22]. The current magnitude was also higher compared to that of Kenyan study which was 39% [23] and other study conducted in eastern Ethiopia which showed prevalence of 34.4% [24, 25]. Similarly, it was higher than those of studies conducted in southwest Ethiopia, which was 35.4% [26]. A studies in Libo-kemkem and Fogera districts of northwest Ethiopia, and Haramaya district of eastern Ethiopia reported a higher prevalence of 42.7% [27] and 45.8% [28] stunting among school age children, respectively.

Our findings might vary in part from previous ones due to differences in geographic characteristics of the study area [23–25], study period, age difference of the study participants [27] (i.e. 0–59 and 6–59 months) and other socio-economic characteristics of the participants. Higher prevalence of infectious diseases like malaria and Visceral leishmaniosis and micronutrient deficiencies in Libo-kemkem district with inadequate health care may contribute higher occurrences of child stunting in our study subjects [29, 30]. However, the magnitude of stunting in the study area is much higher compared to the national recommendations and efforts to alleviate the problem. For example, the prevalence is consistent with the 2005 national report [31]; however, there have been improvements over that time as reported in 2014 [8].

Also this study showed that the prevalence of stunting increases with the age of the child. This association was supported by other studies in north and northwest Ethiopia [27, 32]. This might be due to the nutritional status of the mother since stunting has a chronic and cyclic nature, poor dietary practice, weaning, lower and inappropriate breast and complementary feeding practices. The other possible explanation for increased risk of stunting in older children may be due to unhygienic preparation of complementary foods which exposes children to recurrent infections. Limited access to safe drinking water in the study area also exposes these children to varied types of infections and diarrheal diseases which further increase the risk of chronic malnutrition.

Our results shows that, education is the key resource that enabled women and men to provide appropriate childcare with regard to health, child feeding and child education. Completion of secondary education of the father was observed to ameliorate the prevalence of stunting among the study participants. These associations were not observed in those completed primary education and may be due to the fact that life science courses are not integrated with nutrition education and communication. Similar associations were seen in studies conducted in Bangladesh and the Philippines [33, 34]. This is because in the study area fathers who are educated better than their wife’s as, household heads have control over family expenditures. Thus, they have a leading role in providing quality health care and optimal feeding for their children. Therefore, if the father is educated, he is more knowledgeable in childcare as well as optimal child feeding recommendations and can advise the mother on children’s nutritional requirements.

On the other hand, this study identified that as family size increases, so to do the odds of being stunted. Children from families with six and more members had a higher odds of being stunted compared to children from five or less family members. This finding is supported by another study conducted in southeast Ethiopia which stated that children whose mothers gave birth to more than four children were more likely to be stunted compared to children from mothers who gave birth to one child[14]. This could be due to the fact that, families with more children are more stretched economically and cannot feed themselves well and face difficulty in providing the daily nutrition requirements for proper child physical development. This means, as the size of a family increases there is a scarcity of resources for household consumption, especially food, and healthcare which ultimately leads to stunted growth. Furthermore, parents with more children generally lack adequate time to pay proper attention to the need of each child.

The occupational status of the household head also has a significant role in a child’s stunting. Households headed by farmers and self-employed parents reduced the odds of stunting among their children compared to households led by housewives. This is because the income earned by a single parent (a mothers) is always often less than what couples can procure.

The study has the following limitations. We cannot declare a temporal relationship between stunting and other independent variables due to the cross sectional design of the study. Standard procedures were used for the measurement of height/length but measurement errors are inevitable especially within assessors. Moreover, there may be a recall bias in reporting age of children in a rural villages.

## Conclusion

Our findings demonstrate a higher prevalence of stunting in Libo-kemkem district and thus represents an important public health concern. This study also revealed that a child’s age, occupational status of the household head, family size, and fathers’ education were significantly associated factors for stunting. Therefore, a strong nutrition specific and sensitive intervention should be implemented in the study area with a special focus on supporting housewives, promoting family planning, and education on child feeding and nutrition.

## Supporting information

S1 Dataset Metadata. (DTA)

## Abbreviations

AOR: Adjusted Odds Ratio
ARI: Acute respiratory infection
CBN: Community Based Nutrition
CI: Confidence Interval
CSA: Central Statistics Agency
COR: Crude Odds Ratio
ENA: Emergency Nutrition Assessment
EDHS: Ethiopian Demographic Health Data
SD: Standard deviation
SMART: Standardize Monitoring and assessment of Relief and Transition
SPSS: Statistical Package for Social Science
WHO: World Health Organization
